# Acceptability of 4-poster deer treatment devices for community-wide tick control among residents of high Lyme disease incidence counties in Connecticut and New York, USA

**DOI:** 10.1016/j.ttbdis.2023.102231

**Published:** 2023-07-31

**Authors:** Courtney C. Nawrocki, Nicholas Piedmonte, Sara A. Niesobecki, Adam Rowe, AmberJean P. Hansen, Alison Kaufman, Erik Foster, James I. Meek, Linda Niccolai, Jennifer White, Bryon Backenson, Lars Eisen, Sarah A. Hook, Neeta P. Connally, Victoria L. Hornbostel, Alison F. Hinckley

**Affiliations:** a Division of Vector-Borne Diseases, National Center for Emerging and Zoonotic Diseases, Centers for Disease Control and Prevention, Fort Collins, CO, USA; b Bureau of Communicable Disease Control, New York State Department of Health, Albany, NY, USA; c Connecticut Emerging Infections Program, Yale School of Public Health, New Haven, CT, USA; d Department of Biology, Western Connecticut State University, Danbury, CT, USA

**Keywords:** Lyme disease, Tick-borne disease, Prevention, Ticks, Humans

## Abstract

The 4-Poster Tick Control Deer Feeder (4-poster) device applies acaricide to white-tailed deer (*Odocoileus virginianus)* and can reduce populations of the blacklegged tick (*Ixodes scapularis*), which transmits the agents of Lyme disease, anaplasmosis, babesiosis, and Powassan virus disease in the Northeastern United States. While 4-poster devices have the potential to provide community-wide management of blacklegged ticks in Lyme disease endemic areas, no recent study has assessed their acceptability among residents. We conducted a survey of residents from 16 counties with high annual average Lyme disease incidence (≥ 10 cases per 100,000 persons between 2013 and 2017) in Connecticut and New York to understand perceptions and experiences related to tickborne diseases, support or concerns for placement of 4-poster devices in their community, and opinions on which entities should be responsible for tick control on private properties. Overall, 37% of 1652 respondents (5.5% response rate) would support placement of a 4-poster device on their own property, 71% would support placement on other private land in their community, and 90% would support placement on public land. Respondents who were male, rented their property, resided on larger properties, or were very or extremely concerned about encountering ticks on their property were each more likely to support placement of 4-poster devices on their own property. The primary reason for not supporting placement of a 4-poster device on one’s own property was the need for weekly service visits from pest control professionals, whereas the top reason for not supporting placement on other land (private or public) was safety concerns. Most respondents (61%) felt property owners should be responsible for tick control on private properties. Communities considering 4-poster devices as part of a tick management strategy should consider targeting owners of larger properties and placing devices on public lands.

## 1. Introduction

Lyme disease is the most common vector-borne disease in the United States, with an estimated 476,000 persons diagnosed per year (Kugeler et al., 2021). Most cases occur in the Upper Midwest, Northeastern, and Mid-Atlantic regions of the country. The blacklegged tick, *Ixodes scapularis*, is the primary vector of *Borrelia burgdorferi* sensu stricto, the bacterium that causes Lyme disease in the Northeastern U.S., as well as other agents causing anaplasmosis, babesiosis, Powassan virus disease, and relapsing fever (Schwartz et al., 2017). Currently available methods for Lyme disease prevention fall into two categories: personal protection and environmental tick control (Eisen, 2020, 2021; Schwartz et al., 2022). Environmental tick control methods include, among others, broadcast application of acaricides or fungal control agents to kill host-seeking ticks, and treatment of animals serving as key hosts for *I. scapularis* with topical acaricides to kill feeding ticks. In most cases, implementation of environmental tick control is the responsibility of the household, and barriers such as cost, lack of awareness of tickborne disease risk, and tolerance for pesticides can lead to sporadic practice throughout communities (Gould et al., 2008; Piesman and Eisen, 2008). Among these methods, deer-targeted approaches, by virtue of the large home range of white-tailed deer (*Odocoileus virginianus*), their abundance in suburban neighborhoods, and the fact that they are the primary reproductive host for female blacklegged ticks, have strong potential to provide community-wide suppression of *I. scapularis* (Eisen and Stafford, 2021).

The 4-Poster Tick Control Deer Feeder (4-poster) device, a patent of the U.S. Department of Agriculture’s Agricultural Research Service, was developed to topically treat white-tailed deer with acaricide to control deer-feeding ticks such as *I. scapularis* and the lone star tick, *Amblyomma americanum* (Pound et al., 2009a). The 4-poster device consists of four vertical, acaricide-impregnated applicator rollers, two each on either side of a central bait bin filled with whole kernel corn. As deer consume the corn, the sides of the head, neck, and ears, where ticks commonly feed, rub against the applicators and are treated with a permethrin-based acaricide (4-Poster Tickicide containing 10% permethrin, Y-Tex Corporation, Cody, WY, USA) approved by the Environmental Protection Agency (EPA) for use with 4-poster devices (Environmental Protection Agency, 2021).

4-Poster devices primarily target *I. scapularis* adults by interrupting tick reproduction. Due to the multi-year life cycle of *I. scapularis*, significant reductions in the abundance of nymphs, the life stage considered to account for most human infections with *I. scapularis*-associated pathogens, are not expected to occur until at least two years post-deployment (Pound et al., 2009a). For this reason, devices must be operated for several consecutive years to achieve suppression of *I. scapularis* nymphs and must remain in operation thereafter during seasons of peak tick activity for continuous control. Previous studies involving high-density placement of devices (one device per 20–25 ha) have shown them to be effective in reducing host-seeking *I. scapularis* nymphs on 5 km^2^ treatment sites by about 50% in the third year of use and about 60% and 70% in the fourth and sixth years, respectively (Brei et al., 2009; Curtis et al., 2011; Pound et al., 2009b).

Federal and state-specific regulations place limits on use of 4-poster devices. The EPA requires that Tickicide, which is approved for use with 4-poster devices, be applied by licensed pest control professionals. Additionally, warning signs must be placed around each device, and if a device is placed within 91 m (300 ft) of a home or place where children may be present, a 71–76 cm (28–30 in) high protective fence must surround it. The state of New York (NY) has imposed additional restrictions for use of 4-poster devices, including requiring that a deer feeding permit be obtained prior to placement, prohibiting device placement within 91 m (300 ft) of a public road or highway, and requiring consent from all owners of properties that fall at least partially within a 16 ha (40 ac) area surrounding a proposed device location (i.e., within a radius of 227 m, or 745 ft, from the device) (New York State Department of Environmental Conservation, 2021). Additionally, in areas of NY where bears are a local concern, electric fencing around devices may be required.

The potential for 4-poster devices to reduce human exposure to *I. scapularis* in residential settings, where human interactions with this species frequently occur (Falco and Fish, 1988; Fischhoff et al., 2019; Jordan and Egizi, 2019; Mead et al., 2018; Williams et al., 2018), can be limited by the willingness of residents to host devices on their property or support placement of devices on other lands in their community. Understanding the acceptability of 4-poster devices as a method for community-wide control of blacklegged ticks, and reasons for lack of acceptance among residents, is critical for Lyme disease endemic communities considering implementing 4-poster devices as part of a tick management strategy. We aimed to evaluate the acceptability of using 4-poster devices among residents of high Lyme disease incidence areas in Connecticut (CT) and NY by assessing willingness of respondents to have a 4-poster device placed on their properties and in their communities, and identifying characteristics associated with, and reasons for, lack of support. This work was carried out along with efforts to understand the logistical feasibility of conducting an intervention trial to assess the impact of 4-poster devices on human-tick encounters in residential settings (Connally et al., 2022).

## 2. Materials and methods

### 2.1. Study design, population, and sampling

We conducted a cross-sectional, population-based survey of residents in selected high Lyme disease incidence counties in CT and NY during April-May of 2021. High Lyme disease incidence counties were defined as counties with a five-year (2013–2017) average Lyme disease incidence ≥ 10 cases per 100,000 persons (Schwartz et al., 2017). Our target population included residents of all eight counties in CT, and eight selected counties in NY (Appendix A). We stratified by state to allow for comparisons between CT and NY. Additionally, because one NY county (Suffolk County) had previously deployed 4-poster devices as a form of tick management (Curtis et al., 2011), we aimed to have sufficient sample size in NY to allow for county-level comparisons to be made based on residents’ previous exposure to 4-poster devices in their communities. The number of households targeted per county was proportional to county population.

Because acceptability of using 4-poster devices to control ticks on deer has not been recently assessed and a previous assessment of acceptability of using pesticides to control ticks on deer (Gould et al., 2008) did not specify placement locations of deer treatment devices, we based our sample size calculations on the conservative estimate that 50% of respondents would be likely to support placement of a 4-poster device on their own property. Application of these parameters, alpha equal to 0.01, and an acceptable error rate of +/− 5% yielded a required sample size of 1500 respondents, including 500 respondents from CT and 1000 respondents from NY (500 respondents from Suffolk County and 500 respondents pooled across the other seven NY counties, hereafter referred to as the Hudson Valley counties).

Based on the above-mentioned sample size calculations and an anticipated response rate of 5% (Niesobecki et al., 2019), we identified a random selection of 10,000 addresses in CT and 20,000 in NY (10,000 in Suffolk County and 10,000 pooled across the Hudson Valley counties) for recruitment, for a total of 30,000 addresses. Residential addresses were purchased from Marketing Systems Group (MSG) (Horsham, PA, USA), a marketing company that receives address lists from the United States Postal Service. Lists are updated monthly based on change of address submissions. Seasonal residences, educational residences, vacant properties, and Post Office Boxes were excluded from sampling in both states; single receptacles serving multiple residences (“drop sites”) were included in NY when available and excluded in CT. Additionally, we purchased an indicator of urbanicity for each address, which MSG designates based on several factors, including census block (Marketing Systems Group, 2022).

### 2.2. Recruitment and enrollment

Recruitment postcards were sent to identified addresses in both states in April of 2021. Inclusion criteria required that a participant should be a member of the household to which the postcard was mailed and aged 18 years or older. Postcards included a brief description of the survey, eligibility criteria, instructions on how to take the survey, information on compensation, and a link to the electronic survey (via a typed link as well as a scannable Quick Response code) with a unique access code linked to the respondent’s address. Respondents were also given the option to take the survey by phone with a study site coordinator if preferred. The survey remained open for approximately 30 days. A reminder postcard was sent two weeks after the initial postcard. Respondents were mailed a thank you letter and a $10 gift card after completion of the survey.

### 2.3. Data collection

Survey data were collected using Research Electronic Data Capture (REDCap) software (Vanderbilt University, Nashville, TN, USA) hosted by Yale University, New Haven, CT. Before answering questions about 4-poster devices, respondents were given the option to learn about the devices by either watching a short, informational video embedded in the survey, or reading the same information. The NY version of the survey included information about the additional NY-specific requirements for 4-poster placement (e.g., electric fencing is required in areas where bears may be present). Survey questions included demographic characteristics, respondent concerns and experiences related to tickborne diseases, history of tickborne disease diagnosis among household members, what entities should be responsible for tick control on private properties, whether the respondent had ever heard of 4-poster devices prior to taking the survey, and whether respondents would support placement of a 4-poster device on their own property, other private properties in their neighborhood, or public lands in their community (see Supplementary Material 1). Respondents who answered “No” or “Unsure” to whether they would support placement of 4-poster devices on any of the three property types were then asked to select reasons for lack of support or for being unsure. Respondent sex and other demographics were defined based on self-report. Respondents that chose to take the survey by phone were read the information by a study site coordinator.

The study protocol was reviewed and approved by Institutional Review Boards and ethics committees at the Centers for Disease Control and Prevention, Connecticut Department of Public Health, Yale University, and the New York State Department of Health. Respondents were informed about the nature and purpose of the survey through the recruitment postcard and survey introduction; deliberate navigation to the survey link and subsequent completion of the survey demonstrated consent to participate.

### 2.4. Data analysis

Survey weights were calculated by multiplying the inverse of the number of households surveyed per county by the total number of households available to be sampled in the county (Lumley, 2004; Fowler, 2014) based on estimates provided by MSG. Weights were applied in all analyses to account for household selection probability. Descriptive statistics were generated to describe respondent and household characteristics, experiences with and attitudes towards tickborne diseases and 4-poster devices, opinions on who should be responsible for tick control on private properties, and concerns regarding placement of 4-poster devices. All reported frequencies are unweighted and proportions are weighted. Pearson’s chi-square tests were used to compare responses by state and by county-level exposure to 4-poster devices within NY. Multivariable logistic regression models were used to evaluate whether certain characteristics were associated with supporting placement of 4-poster devices in each of the three scenarios (i.e., on the respondent’s own property, on other private property in their neighborhood or community, or on other public land in their neighborhood or community), while adjusting for covariates, including respondent demographics, household and property characteristics, perceptions and experiences related to tickborne diseases, and ever having heard of 4-poster devices. Alpha was set at 0.05 for all statistical tests of significance. All analyzes were conducted in R 4.0.2 (Core Team, 2018). Tidyverse packages were used to clean and organize data (Grolemund and Wickham, 2011; Wickham, 2017, 2018; Wickham et al., 2018a,b), and the srvyr package (Freedman Ellis and Schneider, 2021) was used to incorporate calculated weights in analyses.

## 3. Results

We received a total of 1652 surveys (543 from CT and 1109 from NY) for an overall survey response rate of 5.5% (5.4% in CT and 5.5% in NY). Across both study sites, 56% of respondents were female, and 62% were aged 50 years or older. The majority (64%) of respondents had a property size < 0.4 ha (1 ac), lived in a suburban area (69%), and did not live in a household with a child under the age of 18 years (68%). While 98% of respondents confirmed via a question in the survey that they did either watch or read the information about 4-poster devices before answering the questions, more respondents chose to watch the video (54%) compared to read the information (44%). Compared to NY, respondents in CT were more often male, had larger property sizes, and lived in a rural or urban (versus suburban) area. Respondents in NY more often had children living in the household (Table 1).

Within NY, we received 539 surveys from Suffolk County (5.4% response rate) and 570 from the Hudson Valley counties (5.7% response rate). Respondent and household characteristics for the Hudson Valley counties and Suffolk County are presented in Appendix B.

### 3.1. Experiences with and attitudes towards tickborne diseases and 4-poster devices

Across CT and NY, approximately 30% of respondents reported that they or someone else in their household had ever been diagnosed with a tickborne disease by a healthcare provider; of these, 47% reported they were diagnosed more than five years ago, 39% reported they were diagnosed between one and five years ago, and 13% reported they were diagnosed in the past year (Table 2). More than half of respondents were very or extremely concerned that they or another household member would encounter ticks both while spending time on their own property (55%) and in their neighborhood or community (61%). Additionally, more than half of respondents were very or extremely concerned that they or another household member would get a tickborne disease while spending time on their own property (52%) and in their neighborhood or community (57%). Compared to NY, more respondents from CT were at least somewhat concerned they or someone else in their household would get a tickborne disease while spending time on their own property.

Only 6% of respondents had ever heard of 4-poster devices, with more respondents from NY having heard of them (8%) compared to CT (3%) (Table 2). Overall, 37% of respondents stated that they would support placement of a 4-poster device on their own property, with support being higher in CT (42%) compared to NY (34%). Seventy-one percent of respondents indicated that they would support placement of a 4-poster device on other private land in their neighborhood or community, with support being similar in NY (70%) and CT (74%). Ninety percent of respondents stated that they would support placement of a 4-poster device on public land in their neighborhood or community, with support again being similar in NY (91%) and CT (89%).

Respondent and household experiences with and attitudes towards tickborne disease and 4-poster devices for the Hudson Valley counties and Suffolk County are shown in Appendix C. Notably, a larger proportion of respondents had heard of 4-poster devices in Suffolk County (13%) compared to the Hudson Valley counties (3%), and a smaller proportion of respondents would support placement of a 4-poster device on their own property in Suffolk County (29%) compared to the Hudson Valley counties (39%).

### 3.2. Characteristics associated with support for placement of devices

Unadjusted and adjusted odds ratios for selected characteristics and support for placement of devices are shown in Table 3. In multivariable logistic regression analysis, respondent characteristics associated with supporting placement of a device on one’s own property were being male, renting the property they lived on, living on a property ≥ 0.2 ha (0.5 ac), and being very or extremely concerned about encountering ticks on their own property. Having children living in the household was not associated with support for placement of devices on one’s own property. Characteristics associated with supporting placement of a device on other private property and public land in one’s neighborhood or community were similar to on one’s own property. Ever having heard of 4-poster devices before taking the survey was not associated with support for placement of devices in any of the three scenarios.

Unadjusted and adjusted odds ratios for selected characteristics and support for placement of devices for the Hudson Valley counties and Suffolk County are shown in Appendix D. Region of NY (i.e., Suffolk County versus Hudson Valley counties) and ever having heard of 4-poster devices before taking the survey were not associated with support for placement of devices in any of the three scenarios.

### 3.3. Reasons for lack of support for placement of 4-poster devices

Among those who answered they would not or were unsure if they would support placement of a device on their own property, the top reason was weekly visits from pest control professionals to service the devices (35%), followed by damage to property or landscape from deer or other wildlife (30%), not thinking this would apply to where they live (29%), and safety of the devices (26%) (Table 4). Among those who selected safety of the devices, specific concerns were safety of pesticides for the respondent, their family, or pets (80%), followed by safety of pesticides for the environment (55%), safety related to attracting wildlife (48%), and safety of pesticides for deer and other wildlife (40%). Women were significantly more likely than men to answer that they were not concerned about ticks on their own property (*p* = 0.01), that they had concerns about safety of the devices (*p* = 0.003), and that they needed more information on how the devices work (*p* = 0.04) (data not shown).

Among those who answered they would not or were unsure if they would support placement of a device on other private property or public land in their neighborhood or community, the top reason was safety of the devices (31% for other private property and 48% for public land). Among those who selected safety as a concern in both of these placement scenarios, specific concerns most often selected pertained to safety of pesticides for the respondent, their family, or pets (74% for other private property and 69% for public land) and safety of pesticides for the environment (62% for other private property and 71% for public land). Damage to property or landscape from deer or wildlife was also a significant concern among those who would not or were unsure if they would support placement of a device on other private land in their neighborhood or community (25%). Women were again significantly more likely than men to have safety concerns about devices placed on other private property in their neighborhood or community (*p* = 0.002) (data not shown).

Compared to NY respondents, CT respondents were more likely to select “how the device looks” as a reason for lack of support for placement of devices on their own property (*p* = 0.01), and also more likely to select “need more information on how the device works” as a reason for lack of support for placement on other private land in their neighborhood or community (*p* = 0.02) (data not shown).

Within NY, compared to the Hudson Valley respondents, Suffolk County respondents were significantly more likely to select that they were “not worried about ticks in this environment” (*p* < 0.001), that they use other methods of tick control (*p* = 0.03), and that they do not think this applies to where they live (*p* < 0.001) for placement of 4-poster devices on their own property (data not shown). For placement of 4-poster devices on other private property in their neighborhood or community, Suffolk County respondents were significantly more likely to select they were “not worried about ticks in this environment” (*p* < 0.01) and do not think this applies to where they live (*p* < 0.001) (data not shown).

### 3.4. Responsibility for tick control on private properties

**Fig. 1.**
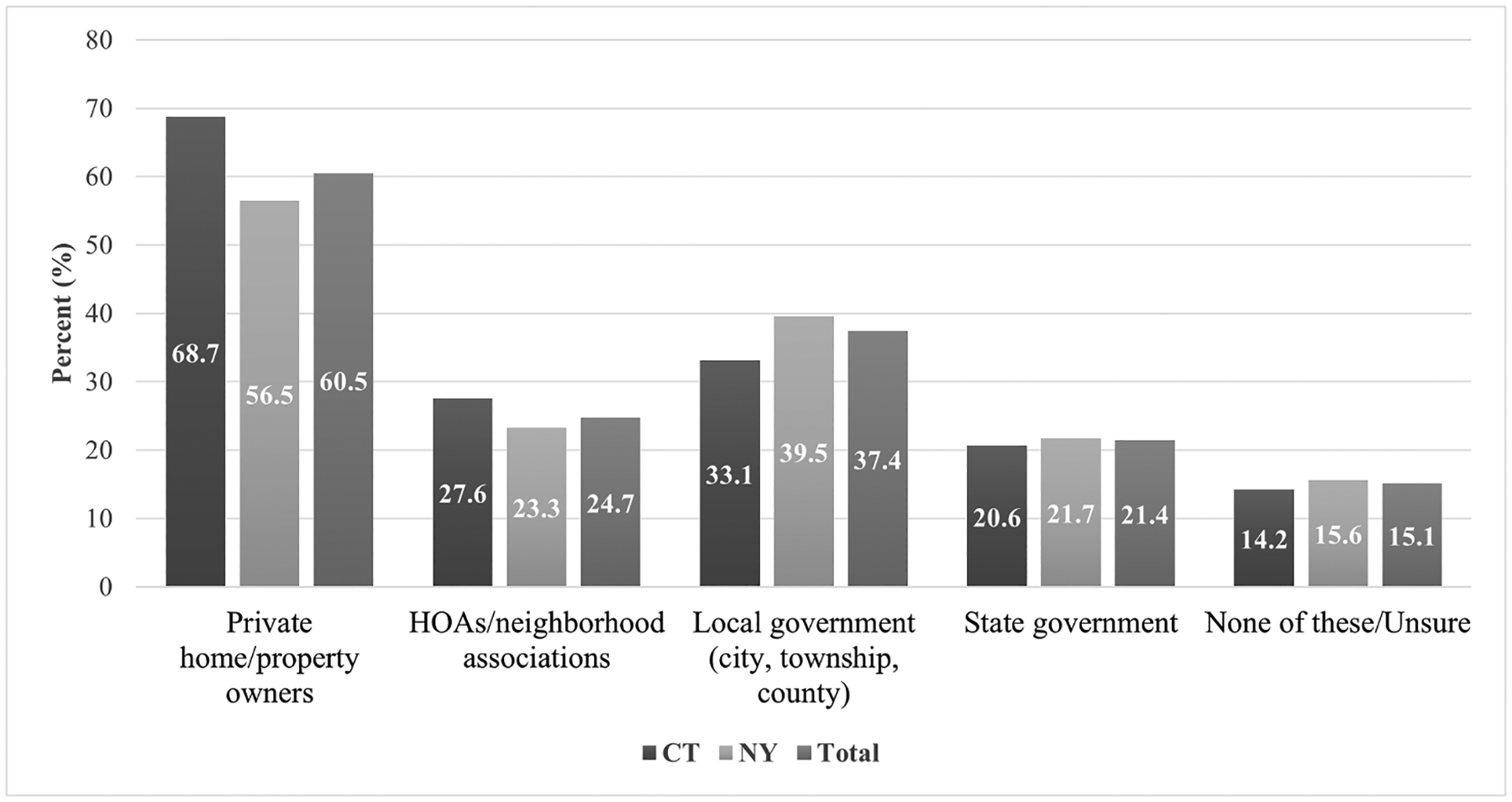
Respondent opinion on who should be responsible for tick control on private properties, Connecticut and New York, USA, 2021. *Denotes statistically significant differences between states. **Multiple answers were allowed; totals may exceed 100%.

The most common response for who should be responsible for tick control on private properties was private homeowners and property owners (61%), followed by local government (e.g., city, township, or county) (37%), homeowner’s associations and other neighborhood associations (25%), and state government (21%) (Fig. 1). More respondents from CT felt that private homeowners and property owners should be responsible for tick control on private properties compared to NY (*p* < 0.001), and more respondents from NY felt that local government should be responsible for tick control on private properties compared to CT (*p* = 0.01) (data not shown).

Within NY, more respondents from Suffolk County (43%) felt that local government should be responsible for tick control on private properties compared to Hudson Valley (36%) (*p* = 0.02) (data not shown).

## 4. Discussion

In this study, we found that most respondents supported placement of 4-poster devices in their communities, though a smaller proportion supported placement of a device on their own property. Support for placement on other private and public community lands was similar to a CT study in which approval of using pesticides on deer as a community intervention to control Lyme disease ranged from 64 to 80% in three health districts in 2002 and 2004 (Gould et al., 2008). Comparatively lower acceptability of hosting a device on one’s own property in our study could be partially explained by the finding that more respondents were “very or extremely concerned” about both encountering ticks and getting a tickborne disease while spending time in their neighborhood or community compared to while spending time on their own property. Respondents, especially those that take tick control measures on their own property, may feel more protected there than they do on other community lands, despite evidence that most tick encounters occur on private property controlled by the respective homeowner in suburban areas of the Northeastern U.S. (Mead et al., 2018).

CT respondents were significantly more likely to support placement of a device on their own property compared to NY respondents, although state was not associated with support in the multivariable logistic regression model. However, CT respondents were significantly more likely to be male and have larger property sizes, both of which were associated with supporting placement of a 4-poster device on one’s own property. Larger properties could facilitate placement of a device away from the home and in less visible places, and therefore these respondents may have been less likely to have concerns about how the device looks. Previous studies have found that larger property size is associated with increased risk for tick encounters (Hook et al., 2021; Maupin et al., 1991), likely because larger properties more often border or contain tick habitat compared to smaller properties. Thus, larger property sizes in CT could also explain why CT respondents were more concerned about getting a tickborne disease while spending time on their own property compared to NY respondents.

Concern for encountering a tick while spending time on one’s own property was found to be associated with support for placement of a device in this setting. Knowledge, perceived severity, and risk of Lyme or other tickborne diseases have previously been found to influence willingness to use personal protection and environmental tick control measures (Beck et al., 2022; Gould et al., 2008; Herrington et al., 1997; Niesobecki et al.,2019; Shadick et al., 1997).

While it is possible Suffolk County’s previous experience with community 4-poster devices could be contributing to NY respondents being overall less likely to support placement of a 4-poster device on their own property, ever having heard of these devices prior to taking the survey was not associated with support for device placement. Suffolk County respondents were significantly less likely to support placement of a device on their own property compared to Hudson Valley respondents, but it is possible this difference was driven by smaller property sizes, fewer households having ever experienced a tickborne disease, and less concern for getting a tickborne disease on one’s own property in Suffolk County.

Our results indicate that lack of support for placement of a 4-poster device on one’s own property is driven, in part, by concerns related to property and landscape damage from deer or other wildlife visiting the devices, and weekly visits from pest management professionals. Several studies have documented heavy non-target animal use of devices, primarily by squirrels (*Sciurus carolinensis*) and raccoons (*Procyon lotor*) (Curtis et al., 2009, 2011; Edwards et al., 2016; Grear et al., 2014;Harmon et al., 2011; Wong et al., 2016; V. Horbostel, personal communication), rendering potential damage resulting from attraction of these animals to the property a valid concern. Weekly visits from pest management professionals are required to re-apply acaricide to the rollers, replenish corn bait that has been consumed by deer and non-target animals, readjust devices as needed, and repair any damage. One pilot study that deployed 4-poster devices on a golf course in CT also reported some property damage upon removal of the devices, particularly holes from animal digging and bare ground where the devices themselves sat (Curtis et al., 2011; V. Hornbostel, personal communication).

Despite high support for placement of devices on other private and public land in one’s neighborhood or community, safety of the devices—and particularly, safety of pesticides—was the top reason cited among those who did not support or were unsure if they would support placement of a device in these two settings. Concerns related to safety of pesticides and hesitancy to use them as a tick control method have been described in other studies (Beck et al., 2022; Gould et al., 2008; Hooket al., 2015; Niesobecki et al., 2019), and are particularly high in New England (Hook et al., 2015), although these studies mostly refer to broadcast spraying of acaricides into the environment, which presents a different set of risks than 4-posters. While some people may be unwilling to use any form of synthetic pesticide to control ticks on their properties, and while the pesticide used with 4-poster devices is not contained within the device and therefore may cause more concern for family members and pets than for rodent-targeted devices where the pesticide is contained within tubes or boxes, it is important to note one of the benefits of 4-poster devices is that they do not disseminate pesticides widely into the environment. Communities interested in using 4-poster devices as part of a community tick control program should ensure this information is communicated to residents, as well as the requirement of a protective fence when placement is in areas close to a home or place children may be present.

Interestingly, being male was associated with support for placement of a device in all three scenarios, and women were more likely to have safety concerns about placement of devices on their own property and other private property in their neighborhoods and communities. A review of individual and social factors that influence pro-environmental concern found that women may have stronger environmental concerns compared to men, which could contribute to the higher levels of concern about the safety of the devices that we observed (Gifford and Nilsson, 2014). Similarly, a survey of CT and Maryland residents found that women had higher odds of applying natural pesticides to their yards compared to men (Niesobecki et al., 2019). Thus, it is possible that women tend to be more concerned about safety, and particularly the safety of chemical pesticides. Providing more information about how the devices work and the pesticide used could increase acceptability and confidence among this group (Hornbostel et al., 2021). Additionally, men spend more time on average working outside on their property doing lawn and garden care (U.S. Bureau of Labor Statistics, 2021), and therefore may be more concerned about ticks in this environment than women.

Although most respondents felt that property owners should be responsible for tick control on private properties, a large proportion of respondents also felt that local government should be responsible. The finding that more respondents from Suffolk County felt local government should be responsible compared to the Hudson Valley counties could be due to their previous experience with 4-posters deployed in their communities as part of a communitywide tick control program implemented by the local government (Curtis et al., 2011). A recent survey of residents of offshore islands of Maine found that most respondents thought the town or state should be responsible for management of tick control on their island (Elias et al., 2021), and a 2019 survey of residents of Michigan, Minnesota, and Wisconsin found that 80% of respondents would be willing to support a county-wide tick control program by paying a $10 increase in household taxes per year if it were to halve the risk of tickborne diseases in their county (Beck et al., 2022). Historically, tick control on private properties has been thought of as the responsibility of the property owner, but there is an increasing call for professionally staffed, community-wide tick management programs as tickborne diseases continue to increase and the geographic distributions of *I. scapularis* and other vector tick species expand (Eisen, 2020). Our findings indicate that overall, respondents in CT and NY may be supportive of efforts to establish and potentially pay taxes to support tick management programs in their communities.

### 4.1. Limitations

There were several limitations to this study. First, compared to the 2020 census population proportions for both states, a larger proportion of our survey respondents were female, older in age, property owners, and had children < 18 years living in the household, indicating our sample may not be representative of the general population in the sampled jurisdictions. Second, our results may reflect a population that is generally more concerned about or interested in tickborne diseases compared to the general population and therefore more likely to respond to a survey about a tick control method, introducing self-selection bias. However, the proportion of respondents who answered that they or someone in their household had ever been diagnosed with a tickborne disease was lower in our study than in another recent tickborne disease prevention study in CT (Niesobecki et al., 2019), indicating self-selection bias may be lower in our study. Third, even though respondents were required to click through information about the devices in order to proceed to the survey questions and were asked whether they read or watched the information, we have no way to confirm that they actually consumed or understood the information presented. Similarly, we were unable to select for a respondent population that resided on properties that would be suitable for device placement and were unable to evaluate accuracy of a respondent’s evaluation of whether the device applies to where they live.

Additionally, due to differences in sampling methodology and the addition of information about electric fencing to prevent bears in the NY version of the survey, results may not be directly comparable between the two states. Lastly, because only select counties were sampled in NY, these results may not be representative of other counties in the state, though these counties were selected based on high Lyme disease incidence and suitability for adoption of 4-poster devices as a method of community tick control.

## 5. Conclusions

Results from this survey showed resident support for placement of devices in communities, and clarified factors associated with support, and reasons for lack of support, in high Lyme disease incidence counties in CT and NY. Our findings suggest that most people would find a community-wide tick management program that includes 4-poster devices acceptable. Hesitancy to place devices on one’s own property could present a barrier to communities identifying an adequate number of locations for a high-density deployment of devices. When looking for locations to deploy devices, communities could consider targeting owners of larger properties or placement of devices on public lands.

We also found that respondents were less concerned about encountering ticks and getting a tickborne disease on their own property compared to in their neighborhood or community, indicating a continued need for increased awareness about tick bite risk and prevention in the peridomestic setting.

## Supplementary Material

Supplementary material

## Figures and Tables

**Table 1:** Respondent and household characteristics, Connecticut and New York, USA, 2021.

Respondent/Household Characteristic	Connecticut (*N* = 543) No. (%)	New York (*N* = 1109) No. (%)	Total (*N* = 1652) No. (%)	P-value^a^
Sex				
Female	280 (51.6)	646 (58.3)	926 (56.1)	0.01
Male	234 (43.1)	404 (36.4)	638 (38.6)	
Age group, years				
18–49	180 (33.1)	382 (34.4)	562 (34.0)	0.74
≥ 50	337 (62.1)	685 (61.8)	1022 (61.9)	
Rent or own property				
Rent	75 (13.8)	128 (11.5)	203 (12.3)	0.28
Own	437 (80.5)	895 (80.7)	1332 (80.6)	
Property Size				
< 0.2 ha^b^	164 (30.2)	415 (37.4)	579 (35.0)	0.001
0.2 to < 0.4 ha	149 (27.4)	321 (28.9)	470 (28.5)	
0.4 to < 0.8 ha	85 (15.7)	125 (11.3)	210 (12.7)	
≥ 0.8 ha	91 (16.8)	131 (11.8)	222 (13.4)	
No yard	25 (4.6)	52 (4.7)	77 (4.7)	
Urbanicity^c^				
Rural	125 (23.0)	171 (19.7)	296 (21.1)	<0.0001
Suburban	332 (61.1)	898 (75.2)	1230 (69.2)	
Urban	86 (15.8)	40 (5.2)	126 (9.7)	
Household size (mean)	2.7	2.8	2.8	0.65^d^
Children (< 18 years) living in household				
Yes	126 (23.2)	314 (28.3)	440 (26.6)	0.03
No	389 (71.6)	738 (66.5)	1127 (68.2)	

a P-value represents comparisons between CT and NY; evaluated using Pearson’s chi-square test.

b Hectare.

c Designations made by Marketing Systems Group.

d Evaluated using a two-sample independent *t*-test.

**Table 2:** Respondent and household experiences with and attitudes towards tickborne diseases and 4-poster devices, Connecticut and New York, USA,

	Connecticut (*N* = 543) No. (%)	New York (*N* = 1109) No. (%)	Total (*N* = 1652) No. (%)	P-value^a^
Anyone in household ever diagnosed with a tickborne diseaseby a healthcare provider				
Yes	170 (31.3)	332 (29.9)	502 (30.4)	0.60
No/Unsure	368 (67.8)	768 (69.3)	1136 (68.8)	
When household member was diagnosed with a tickborne disease^b^				
In the past year	19 (11.2)	47 (14.2)	66 (13.1)	0.34
1–5 years ago	62 (36.5)	134 (40.4)	196 (39.0)	
> 5 years ago	87 (51.2)	149 (44.9)	236 (47.0)	
Concerned that they or another household member will encounter ticks while spending time on their own property				
Very/Extremely	307 (56.5)	597 (53.8)	904 (54.7)	0.37
Slightly/Somewhat	186 (34.3)	390 (35.2)	576 (34.9)	
Not at all	47 (8.7)	118 (10.6)	165 (10.0)	
Concerned that they or another household member will encounter ticks while spending time in their neighborhood or community				
Very/Extremely	338 (62.2)	669 (60.3)	1007 (61.0)	0.52
Slightly/Somewhat	184 (33.9)	389 (35.1)	573 (34.7)	
Not at all	18 (3.3)	48 (4.3)	66 (4.0)	
Concerned that they or another household member will get a tickborne disease while spending time on their own property				
Very/Extremely	287 (52.9)	577 (52.0)	864 (52.3)	0.01
Slightly/Somewhat	212 (39.0)	394 (35.5)	606 (36.7)	
Not at all	39 (7.2)	130 (11.7)	169 (10.2)	
Concerned that they or another household member will get a tickborne disease while spending time in their neighborhood or community				
Very/Extremely	307 (56.5)	635 (57.3)	942 (57.0)	0.70
Slightly/Somewhat	210 (38.7)	416 (37.5)	626 (37.9)	
Not at all	21 (3.9)	52 (4.7)	73 (4.4)	
Ever heard of 4-poster device				
Yes	16 (2.9)	84 (7.6)	100 (6.1)	<0.0001
No/Unsure	520 (95.8)	1014 (91.4)	1534 (92.9)	
Watched 4-poster video or read information				
Watched video	284 (52.3)	612 (55.2)	896 (54.2)	0.35
Read information	247 (45.5)	479 (43.2)	726 (43.9)	
Would support placement of 4-poster device^c^				
on own property	226 (41.6)	378 (34.1)	604 (36.6)	0.003
on other private land in neighborhood or community	399 (73.5)	775 (69.9)	1174 (71.1)	0.09
on public land in neighborhood or community	481 (88.6)	1006 (90.7)	1487 (90.0)	0.30

a P-value represents comparisons between CT and NY; evaluated using Pearson’s chi-square test.

b Of those reporting that a household member has ever been diagnosed with a tickborne disease by a healthcare provider.

c Compared to those who selected “No” or “Unsure”.

**Table 3:** Univariate and multivariable logistic regression results for each placement scenario, Connecticut and New York, USA,2021.

	Support placement on own property		Support placement on other private land in neighborhood or community		Support placement on public land in neighborhood or community	
	uOR^a^	aOR^b^	uOR	aOR	uOR	aOR
State						
CT	reference	reference	reference	reference	reference	reference
NY	0.72 (0.58 – 0.89)	0.88 (0.69 – 1.13)	0.81 (0.64 – 1.03)	0.83 (0.63 – 1.10)	1.24 (0.85 – 1.81)	1.26 (0.81 – 1.96)
Sex						
Female	reference	reference	reference	reference	reference	reference
Male	1.51 (1.23 – 1.86)	1.54 (1.21 – 1.95)	1.28 (1.01 – 1.61)	1.48 (1.12 – 1.95)	1.83 (1.22 – 2.81)	2.79 (1.69 – 4.62)
Age, years						
18–49	reference	reference	reference	reference	reference	reference
≥50	1.14 (0.92 – 1.41)	1.17 (0.87 – 1.56)	0.70 (0.55 – 0.89)	0.80 (0.57 – 1.12)	0.64 (0.42 – 0.97)	0.78 (0.42 – 1.43)
Urbanicity^c^						
Rural	reference	–	reference	–	reference	–
Suburban	0.63 (0.48 – 0.83)	–	0.72 (0.52 – 0.99)	–	1.45 (0.92 – 2.28)	–
Urban	0.80 (0.52 – 1.24)	–	0.71 (0.43 – 1.16)	–	1.31 (0.61 – 2.79)	–
Rent or own property						
Own	reference	reference	reference	reference	reference	reference
Rent	1.77 (1.31 – 2.39)	2.04 (1.40 – 2.95)	1.44 (1.01 – 2.09)	1.47 (0.95 – 2.27)	0.88 (0.52 – 1.59)	0.91 (0.48 – 1.72)
Property size						
< 0.2 ha^d^	reference	reference	reference	reference	reference	reference
0.2 to < 0.4 ha	1.52 (1.14 – 2.02)	1.40 (1.04 – 1.90)	1.34 (1.00 – 1.80)	1.26 (0.91 – 1.74)	1.11 (0.65 – 1.90)	1.13 (0.62–2.07)
0.4 to < 0.8 ha	2.16 (1.52 – 3.06)	1.76 (1.22 – 2.54)	2.06 (1.36 – 3.12)	1.81 (1.17 – 2.81)	0.98 (0.51 – 1.90)	0.80 (0.40 – 1.60)
≥ 0.8 ha	3.25 (2.32 – 4.56)	2.72 (1.88 – 3.93)	1.67 (1.14 – 2.44)	1.64 (1.05 – 2.56)	0.56 (0.33 – 0.97)	0.54 (0.28 – 1.05)
No yard	2.19 (1.32 – 3.62)	1.79 (0.99 – 3.21)	1.19 (0.69 – 2.07)	1.17 (0.64 – 2.16)	0.58 (0.26 – 1.32)	0.57 (0.25 – 1.32)
Children (< 18 years) living in household						
No	reference	reference	reference	reference	reference	reference
Yes	1.12 (0.89 – 1.41)	0.93 (0.69 – 1.26)	0.62 (0.47 – 0.80)	1.39 (0.97 – 1.98)	0.61 (0.37 – 0.95)	1.25 (0.66 – 2.36)
Who should be responsible for tick control^e^						
Private homeowners/property owners	1.34 (1.08 – 1.65)	–	1.29 (1.03 – 1.62)	–	1.42 (0.99 – 2.05)	–
Homeowners/neighborhood associations	1.30 (1.03 – 1.64)	–	1.36 (1.04 – 1.77)	–	1.32 (0.86 – 2.11)	–
Local government (city, township, county)	1.23 (1.00 – 1.51)	–	1.49 (1.18 – 1.89)	–	1.80 (1.20 – 2.75)	–
State government	1.49 (1.17 – 1.90)	–	1.94 (1.45 – 2.64)	–	2.00 (1.20 – 3.55)	–
None of these/Unsure	1.45 (1.08 – 1.95)	–	1.94 (1.46 – 2.58)	–	2.12 (1.37 – 3.21)	–
Concerned that they or another household member will encounter ticks while spending time on their own property						
Very/Extremely	1.78 (1.24 – 2.59)	1.92 (1.22 – 3.04)	2.24 (1.56 – 3.20)	–	1.44 (0.79 – 2.50)	–
Slightly/Somewhat	0.98 (0.67 – 1.45)	1.11 (0.70 – 1.76)	1.18 (0.82 – 1.70)	–	1.26 (0.68 – 2.25)	–
Not at all	reference	reference	reference	–	reference	–
Concerned that they or another household member will encounter ticks while spending time in their neighborhood or community						
Very/Extremely	2.13 (1.21 – 3.93)	–	4.88 (2.89 – 8.31)	4.88 (2.51 – 9.51)	3.07 (1.46 – 5.98)	2.63 (0.99 – 6.95)
Slightly/Somewhat	1.28 (0.72 – 2.40)	–	2.51 (1.48 – 4.30)	2.47 (1.27 – 4.80)	2.20 (1.03 – 4.36)	1.70 (0.65 – 4.47)
Not at all	reference	–	reference	reference	reference	reference
Concerned that they or another household member will get a tickborne disease while spending time on their own property						
Very/Extremely	2.02 (1.41 – 2.94)	–	2.61 (1.82 – 3.73)	–	1.83 (1.05 – 3.08)	–
Slightly/Somewhat	0.98 (0.67 – 1.45)	–	1.20 (0.83 – 1.71)	–	1.66 (0.94 – 2.86)	–
Not at all	reference	–	reference	–	reference	–
Concerned that they or another household member will get a tickborne disease while spending time in their neighborhood or community						
Very/Extremely	1.94 (1.15 – 3.37)	–	3.96 (2.41 – 6.51)	–	2.61 (1.24 – 5.04)	–
Slightly/Somewhat	1.08 (0.63 – 1.90)	–	2.17 (1.31 – 3.57)	–	1.97 (0.93 – 3.86)	–
Not at all	reference	–	reference	–	reference	–
Ever diagnosed with a tickborne disease						
No	reference	–	reference	–	reference	–
Yes	0.81 (0.66 – 1.01)	–	0.83 (0.65 – 1.05)	–	1.27 (0.86 – 1.84)	–
Ever heard of 4-poster devices						
No	reference	reference	reference	reference	reference	reference
Yes	0.96 (0.65 – 1.44)	1.03 (0.62 – 1.69)	1.20 (0.78 – 1.81)	1.12 (0.62 – 2.03)	1.17 (0.56 – 2.20)	0.63 (0.27 – 1.47)

a Unadjusted odds ratio.

b Adjusted odds ratio.

c Designations made by Marketing Systems Group.

d Hectare.

e Multiple options could be selected, so each outcome was evaluated individually. The reference group for each option was not having selected that option.

**Table 4:** Reasons for lack of support for 4-poster devices, among those who would not support placement or were unsure whether they would support placement in each location, Connecticut and New York, USA, 2021.^a.^

	Own property (*N* = 992) No. (%)	Other private land in neighborhood or community^b^ (*N* = 432) No. (%)	Public land in neighborhood or community (*N* = 126) No. (%)
Not worried about ticks in this environment	212 (21.4)	58 (13.4)	9 (7.1)
How the device looks	213 (21.5)	51 (11.8)	8 (6.3)
Safety (general)^c^	260 (26.2)	133 (30.8)	61 (48.4)
Safety of pesticides for self, family, or pets	207 (79.6)	99 (74.4)	42 (68.9)
Safety of pesticides for the environment	143 (55.0)	82 (61.7)	43 (70.5)
Safety of pesticides for deer and other wildlife	104 (40.0)	76 (57.1)	41 (67.2)
Safety related to attracting wildlife	125 (48.1)	66 (49.6)	14 (23.0)
Damage to property/landscape from deer or other wildlife	294 (29.6)	109 (25.2)	–
Weekly visits from pest management professionals	349 (35.2)	–	–
Use other methods of tick control	147 (14.8)	–	–
Feel homeowner’s association or town would not allow it	78 (7.9)	29 (6.7)	6 (4.8)
Need more information on how devices work	94 (9.5)	47 (10.9)	24 (19.0)
Do not think this applies to where they live	291 (29.3)	97 (22.5)	–

a Data presented represents those respondents who answered ‘No’ or ‘Unsure’ when asked whether they would support 4-poster placement in each location listed. Thus, column totals represent different subgroups. Multiple answers were allowed; totals may exceed 100%.

b Some of the reasons for lack of support for placement of devices on one’s own property were not relevant to the other two placement scenarios and thus were not listed as options for those other scenarios (i.e., other private land or public land in neighborhood or community) in the survey.

c Respondents were shown safety concern sub-categories only if they selected “I am concerned about the safety of 4-poster devices”.

## Data Availability

Data will be made available on request.

## Appendix A. Counties targeted for recruitment, Connecticut and New York, USA, 2021

**Table:**

CT (*N* = 8)
Fairfield
Hartford
Litchfield
Middlesex
New Haven
New London
Tolland
Windham
NY (*N* = 8)
Suffolk
Westchester
Rockland
Orange
Putnam
Dutchess
Sullivan
Ulster

## Appendix B Respondent and household characteristics, Hudson Valley counties and Suffolk County, New York, USA, 2021.

**Table:**

Respondent/Household Characteristic	Hudson Valley Counties (*N* = 570) No. (%)	Suffolk County (*N* = 539) No. (%)	Total (*N* = 1109) No. (%)	P-value^a^
Sex				
Female	321 (56.3)	325 (60.3)	646 (58.3)	0.35
Male	216 (37.9)	188 (34.9)	404 (36.4)	
Age group, years				
18–49	199 (34.9)	184 (34.1)	383 (34.5)	0.69
≥50	348 (61.1)	338 (62.7)	686 (61.9)	
Rent or own property				
Rent	83 (14.6)	45 (8.3)	128 (11.5)	< 0.01
Own	436 (76.5)	459 (85.2)	895 (80.7)	
Property Size				
<0.2 ha^b^	161 (28.2)	254 (47.1)	415 (37.4)	< 0.001
0.2 to < 0.4 acre	142 (24.9)	179 (33.2)	321 (28.9)	
0.4 to < 0.8 acres	78 (13.7)	47 (8.7)	125 (11.3)	
≥ 0.8 acres	107 (18.8)	24 (4.5)	131 (11.8)	
No yard	42 (7.4)	10 (1.9)	52 (4.7)	
Urbanicity^d^				
Rural	141 (24.7)	30 (5.6)	171 (15.4)	< 0.001
Suburban	389 (68.2)	509 (94.4)	898 (81.0)	
Urban	40 (7.0)	0 (0.0)	40 (3.6)	
Household size (mean)	2.7	2.9	2.8	< 0.01^c^
Children (<18 years) living in household				
Yes	157 (27.5)	157 (29.1)	314 (28.3)	0.09
No	382 (67.0)	356 (66.0)	738 (66.5)	

a P-value represents comparisons between Hudson Valley counties and Suffolk County; evaluated using Pearson’s chi-square test.

b Evaluated using a two-sample independent *t*-test.

c Hectare.

d Designations made by Marketing Systems Group.

## Appendix C Respondent and household experiences with and attitudes towards tickborne diseases and 4-poster devices, Hudson Valley counties and Suffolk County, New York, USA, 2021.

**Table:**

	Hudson Valley Counties (*N* = 570) No. (%)	Suffolk County (*N* = 539) No. (%)	Total (*N* = 1109) No. (%)	P-value^a^
Anyone in household ever diagnosed with a TBD by a healthcare provider				
Yes	192 (33.7)	140 (26.0)	332 (29.9)	0.03
No/Unsure	371 (65.1)	397 (73.7)	768 (69.3)	
When household member was diagnosed with tickborne disease^b^ In the past year	23 (4.0)	24 (4.5)	47 (4.2)	0.11
1–5 years ago	73 (12.8)	61 (11.3)	134 (12.1)	
> 5 years ago	94 (16.5)	55 (10.2)	149 (13.4)	
Concerned that they or another household member will encounter ticks while spending time on their own property				
Very/Extremely	323 (56.7)	274 (50.8)	597 (53.8)	0.11
Slightly/Somewhat	195 (34.2)	195 (36.2)	390 (35.2)	
Not at all	50 (8.8)	68 (12.6)	118 (10.6)	
Concerned that they or another household member will encounter ticks while spending time in their				
neighborhood or community				
Very/Extremely	350 (61.4)	319 (59.2)	669 (60.3)	0.11
Slightly/Somewhat	201 (35.3)	188 (34.9)	389 (35.1)	
Not at all	17 (3.0)	31 (5.8)	48 (4.3)	
Concerned that they or another household member will get a tickborne disease while spending time on their own property				
Very/Extremely	318 (55.8)	259 (48.1)	577 (52.0)	0.02
Slightly/Somewhat	195 (34.2)	199 (36.9)	394 (35.5)	
Not at all	53 (9.3)	77 (14.3)	130 (11.7)	
Concerned that they or another household member will get a tickborne disease while spending time in their				
neighborhood or community				
Very/Extremely	337 (59.1)	298 (55.3)	635 (57.3)	0.003
Slightly/Somewhat	214 (37.5)	202 (37.5)	416 (37.5)	
Not at all	15 (2.6)	37 (6.9)	52 (4.7)	
Ever heard of 4-poster device				
Yes	16 (2.8)	68 (12.6)	84 (7.6)	<0.001
No/Unsure	546 (95.8)	468 (86.8)	1014 (91.4)	
Watched 4-poster video or read information				
Watched video	323 (56.7)	289 (53.6)	612 (55.2)	0.36
Read information	236 (41.4)	243 (45.1)	479 (43.2)	
Would support placement of 4-poster device^c^ on property	223 (39.1)	155 (28.8)	378 (34.1)	<0.001
on other private land in neighborhood or community	402 (70.5)	373 (69.2)	775 (69.9)	0.09
on public land in neighborhood or community	508 (89.1)	498 (92.4)	1006 (90.7)	0.24

a P-value represents comparisons between Hudson Valley counties and Suffolk County; evaluated using Pearson’s chi-square test.

b Of those reporting that a household member has ever been diagnosed with a tickborne disease by a healthcare provider.

c Compared to those who selected “No” or “Unsure”.

## Appendix D Univariate and multivariable logistic regression results for each placement scenario, Hudson Valley counties and Suffolk County, USA, 2021.

**Table:**

	Support placement on own property		Support placement on other private land in neighborhood or community		Support placement on public land in neighborhood or community	
	uOR^a^	aOR^b^	uOR	aOR	uOR	aOR
Region						
Hudson Valley counties	reference	reference	reference	reference	reference	reference
Suffolk County	0.61 (0.47 – 0.79)	0.84 (0.62 – 1.12)	0.87 (0.67 – 1.14)	1.14 (0.82 – 1.57)	1.37 (0.86 – 2.18)	1.15 (0.65 – 2.03)
Sex						
Female	reference	reference	reference	reference	reference	reference
Male	1.47 (1.11 – 1.95)	1.43 (1.05 – 1.94)	1.22 (0.90 – 1.66)	1.20 (0.85 – 1.69)	1.77 (1.01 – 3.09)	2.02 (1.09 – 3.74)
Age						
18–49	reference	reference	reference	reference	reference	reference
≥50	1.19 (0.89 – 1.58)	1.21 (0.83 – 1.77)	0.77 (0.56 – 1.05)	0.96 (0.63 – 1.46)	0.58 (0.32 – 1.05)	0.50 (0.21 – 1.19)
Urbanicity^c^						
Rural	reference	–	reference	–	reference	–
Suburban	0.53 (0.37 – 0.76)		0.49 (0.32 – 0.77)		1.22 (0.66 – 2.28)	
Urban	0.54 (0.26 – 1.14)	–	0.60 (0.26 – 1.38)	–	1.15 (0.31 – 4.22)	–
Rent or own property						
Own	reference	reference	reference	reference	reference	reference
Rent	1.73 (1.15 – 2.61)	2.06 (1.30 – 3. 26)	2.23 (1.30 – 3.82)	2.12 (1.16 – 3.85)	1.24 (0.53 – 2.93)	1.13 (0.47 – 2.72)
Property size						
< 0.2 ha^d^	reference	reference	reference	reference	reference	reference
0.2 to < 0.4 ha	1.78 (1.25 – 2.54)	1.58 (1.08 – 2.31)	1.41 (0.99 – 2.00)	1.37 (0.92 – 2.02)	1.03 (0.53 – 2.00)	1.19 (0.56 – 2.49)
0.4 to < 0.8 ha	2.60 (1.65 – 4.09)	1.96 (1.20 – 3.19)	1.63 (0.98 – 2.71)	1.48 (0.87 – 2.52)	0.94 (0.40 – 2.24)	0.80 (0.32 – 1.99)
> 0.8 ha	3.71 (2.39 – 5.77)	3.00 (1.84 – 4.90)	2.34 (1.40 – 3.92)	2.79 (1.48 – 5.23)	0.61 (0.30 – 1.26)	0.75 (0.29 – 1.94)
No yard	2.40 (1.28 – 4.48)	2.22 (1.08 – 4.57)	1.77 (0.86 – 3.64)	1.89 (0.88 – 4.06)	0.58 (0.21 – 1.62)	0.47 (0.16 – 1.34)
Children (< 18 years) living in household						
No	reference	reference	reference	reference	reference	reference
Yes	0.96 (0.71 – 1.30)	0.92 (0.62 – 1.36)	1.52 (1.08 – 2.15)	1.35 (0.87 – 2.09)	1.40 (0.76 – 2.58)	0.77 (0.34 – 1.78)
Who should be responsible for tick control^e^						
Private homeowners/property owners	1.36 (1.03 – 1.80)	–	1.27 (0.95 – 1.70)	–	1.52 (0.93 – 2.51)	–
Homeowners/neighborhood associations	1.29 (0.94 – 1.77)	–	1.48 (1.03 – 2.12)	–	1.14 (0.62 – 2.10)	–
Local government (city, township, county)	1.50 (1.14 – 1.98)	–	1.51 (1.11 – 2.04)	–	1.59 (0.93 – 2.72)	–
State government	1.80 (1.31 – 2.49)	–	2.23 (1.50 – 3.31)	–	3.13 (1.48 – 6.65)	–
None of these/Unsure	0.50 (0.10 – 2.41)	–	0.11 (0.02 – 0.55)	–	0.32 (0.07 – 1.39)	–
Concerned that they or another household member will encounter ticks while spending time on their own property						
Very/Extremely	2.13 (1.30 – 3.49)	2.36 (1.27 – 4.38)	1.89 (1.18 – 3.03)	–	1.34 (0.66 – 2.73)	–
Slightly/Somewhat	1.11 (0.65 – 1.87)	1.28 (0.69 – 2.37)	0.92 (0.57 – 1.47)	–	1.62 (0.75 – 3.49)	–
Not at all	reference	reference	reference	–	reference	–
Concerned that they or another household member will encounter ticks while spending time in their neighborhood or community						
Very/Extremely	2.37 (1.08 – 5.24)	–	5.19 (2.62 – 10.31)	4.77 (2.10 – 10.83)	3.03 (1.25 – 7.31)	2.06 (0.69 – 6.18)
Slightly/Somewhat	1.23 (0.55 – 2.77)	–	2.64 (1.32 – 5.29)	2.28 (1.01 – 5.18)	2.80 (1.12 – 6.98)	1.89 (0.61 – 5.89)
Not at all	reference	–	reference	reference	reference	reference
Concerned that they or another household member will get a TBD while spending time on their own property						
Very/Extremely	2.46 (1.51 – 4.01)	–	2.51 (1.59 – 3.94)	–	1.66 (0.85 – 3.26)	–
Slightly/Somewhat	1.22 (0.73 – 2.03)	–	1.06 (0.68 – 1.67)	–	1.74 (0.85 – 3.58)	–
Not at all	reference	–	reference	–	reference	–
Concerned that they or another household member will get a TBD while spending time in their neighborhood or community						
Very/Extremely	2.20 (1.05 – 4.63)	–	5.36 (2.76 – 10.41)	–	2.41 (0.96 – 6.02)	–
Slightly/Somewhat	1.02 (0.47 – 2.18)	–	2.64 (1.35 – 5.14)	–	1.96 (0.77 – 4.97)	–
Not at all	reference	–	reference	–	reference	–
Ever diagnosed with a TBD						
No	reference	–	reference	–	reference	–
Yes	1.25 (0.93 – 1.67)	–	1.16 (0.84 – 1.60)	–	0.68 (0.40 – 1.14)	–
Ever heard of 4-poster devices						
No	reference	reference	reference	reference	reference	reference
Yes	0.79 (0.47 – 1.32)	0.81 (0.46 – 1.42)	0.88 (0.51 – 1.54)	1.26 (0.65 – 2.46)	0.67 (0.27 – 1.64)	0.72 (0.26 – 1.99)

a Unadjusted odds ratio.

b Adjusted odds ratio.

c Designations made by Marketing Systems Group.

d Hectare.

e Multiple options could be selected, so each outcome was evaluated individually. Thus, the reference group for each option was not having selected that option.
