# Supplementary material for: Acceptability of 4-poster deer treatment devices for community-wide tick control among residents of high Lyme disease incidence counties in Connecticut and New York, USA
Source: Ticks Tick Borne Dis. Author manuscript; Available in PMC 2024 Nov 1. (PMC10883357; doi:10.1016/j.ttbdis.2023.102231)
Supplement: Supplementary material [file NIHMS1964690-supplement-Supplementary_material.pdf]

4-Poster Deer Treatment Devices to Reduce Ticks

The U.S. Centers for Disease Control and Prevention, the New York State Department of Health, and the Yale Emerging Infections Program are studying the use of 4-Poster Deer Treatment Devices (or 4-poster devices) to reduce ticks in areas where people live and to possibly reduce tickborne diseases.

This survey will ask about your concerns and experiences with tickborne diseases and views on who should be responsible for tick control on private properties. It will also ask for your thoughts on the use of 4-poster devices to reduce ticks. It is okay if you have not heard of 4-poster devices before now.

This survey will take 10 minutes or less to complete and is completely voluntary. You may go back at any point to change your answers using the buttons at the bottom of the page. You may also leave the survey at any time. If you have questions, problems accessing the survey, or would like to withdraw your responses, please email Sara Niesobecki at ticknet@yale.edu or call 203-764-7949.

Please note: By completing this survey, you are consenting to participate in this survey.

Consent Form

[Attachment: "Consent Form.pdf"]

Are you 18 years of age or older?	◯ Yes<br>◯ No<br>(You must be 18 years of age or older to take this survey and receive a gift card.)

In the coming year, how concerned are you that you, another household member, or your pet could encounter ticks while spending time...

	Not at all	Slightly	Somewhat	Very	Extremely	Prefer not to answer
... on your property?	○	○	○	○	○	○
... in your neighborhood or community?	○	○	○	○	○	○

In the coming year, how concerned are you that you, another household member, or your pet could get a tickborne disease, such as Lyme disease, while spending time...

	Not at all	Slightly	Somewhat	Very	Extremely	Prefer not to answer
... on your property?	○	○	○	○	○	○
... in your neighborhood or community?	○	○	○	○	○	○

Have you or another household member ever been diagnosed with a tickborne disease (such as Lyme disease, anaplasmosis, or babesiosis) by a healthcare provider?	◯ Yes<br>◯ No<br>◯ Unsure<br>◯ Prefer not to answer
When were you or another household member most recently diagnosed with a tickborne disease?	◯ In the past year<br>◯ 1 to 5 years ago<br>◯ More than 5 years ago<br>◯ Unsure<br>◯ Prefer not to answer

Question	Response
Who should be responsible for tick control on private properties?	☐ Private homeowners/property owners<br>☐ Homeowner associations (HOAs)/neighborhood associations<br>☐ Local government (city, township, county)<br>☐ State government<br>☐ None of these<br>☐ Unsure<br>☐ Prefer not to answer<br>(Select all that apply)

This is a photo of a deer feeding at a 4-Poster Deer Treatment Device.

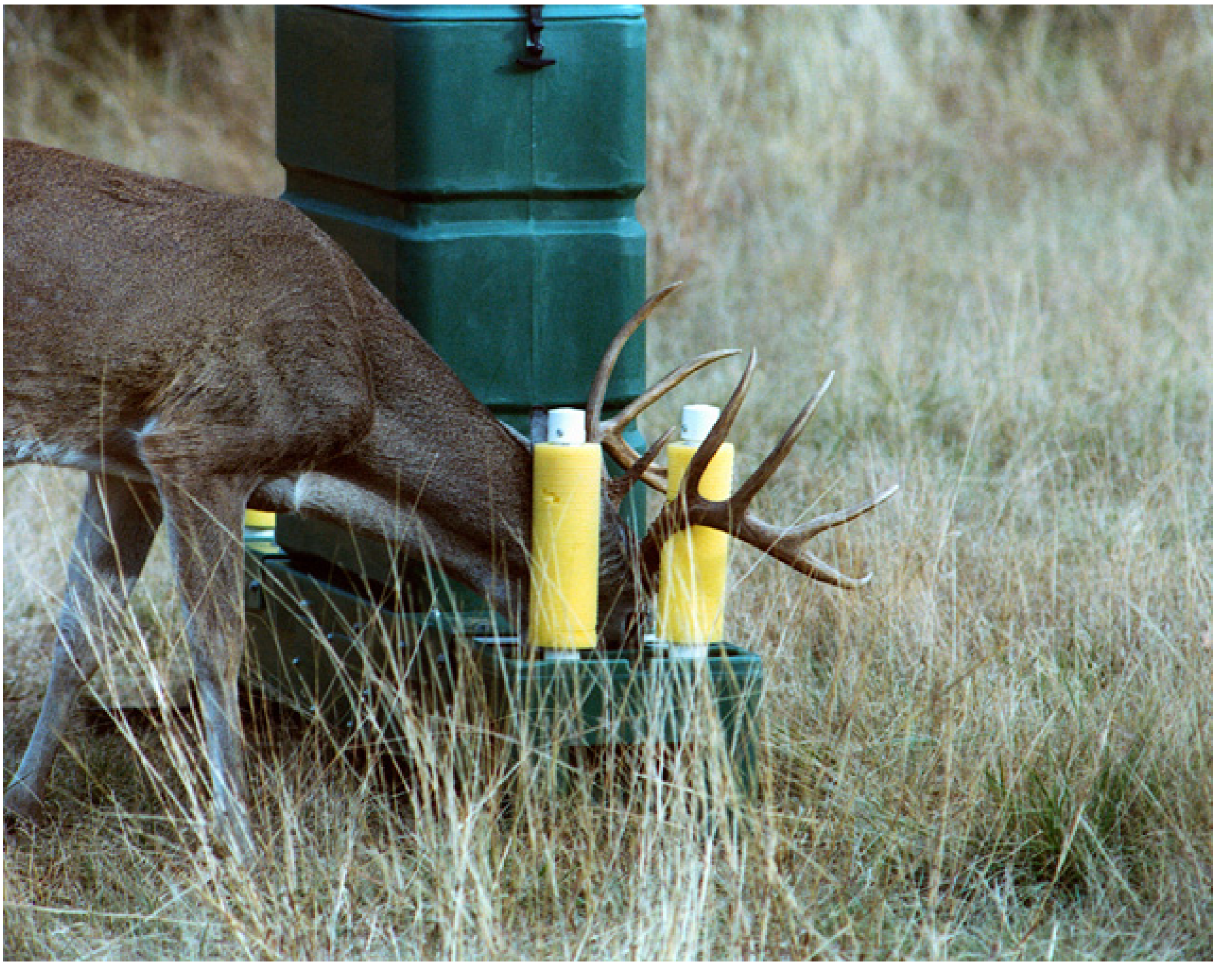

Before taking this survey, had you ever heard of a 4-Poster Deer Treatment Device (or 4-poster device) for reducing ticks?

◯ Yes
◯ No
◯ Unsure
◯ Prefer not to answer

Now, we will give you information on 4-poster devices before asking more questions.

Would you like to watch a short video to learn more about 4-poster devices or read three short pages?

◯ Watch the video
◯ Read three short pages
(If you are using a mobile device, Safari or Google Chrome are recommended to view the video.)

Please click the link below to watch the video. After you have watched the video, please return to the survey and answer the remaining questions.
https://youtu.be/eg7mGafCVas

How the device works (1/3)

Ticks can spread several diseases, including Lyme disease. Deer are important for ticks to reproduce. A 4-poster device treats deer with a pesticide that kills ticks.
4-poster devices have bins filled with corn. As deer eat the corn, they rub against foam rollers ("posts") coated with a pesticide called permethrin that kills ticks.
The pesticide and the device do not harm the deer The treatment is similar to some flea and tick treatments that are applied to the skin of cats and dogs The pesticide does not spread into the environment Devices should not increase deer populations Devices require weekly servicing by licensed professionals

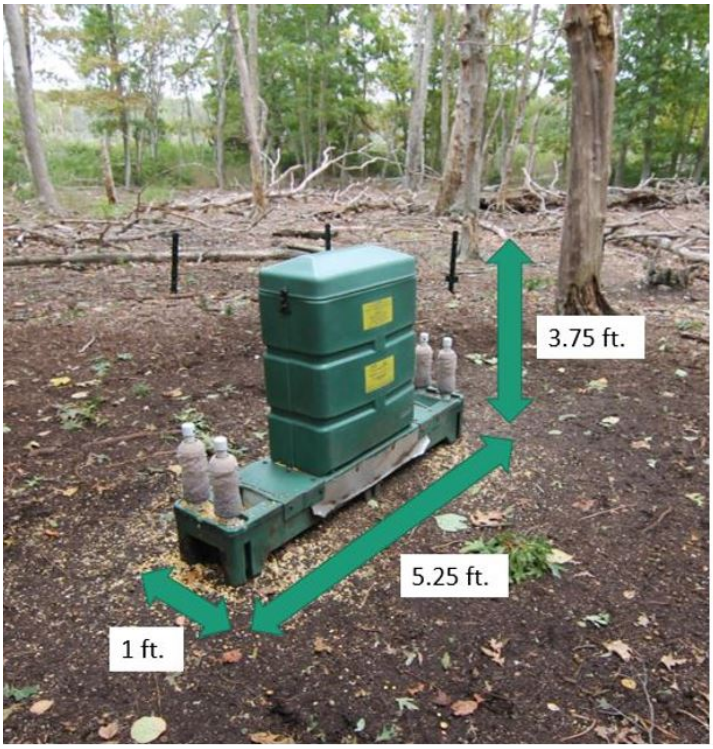

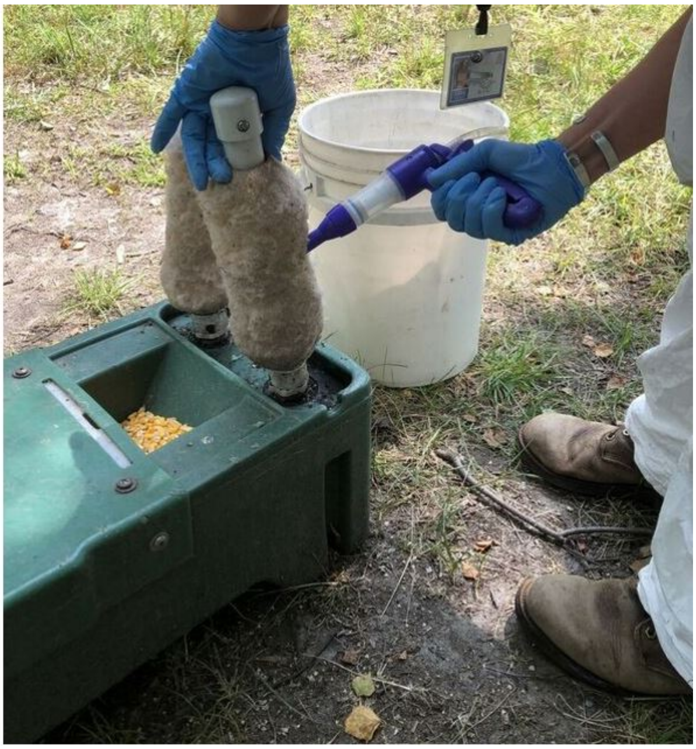

How the device affects ticks (2/3)

4-poster devices are a type of long-term tick management that can be used by a community. Devices need to be in place for 2-3 years to see a decrease in ticks.
Each device will reduce ticks over roughly 50 acres Devices may be used year-round Ticks will eventually return if the devices do not remain in service

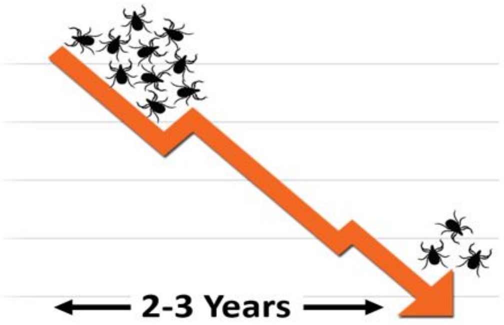

4-poster devices cause the number of ticks in the environment to decrease over time.

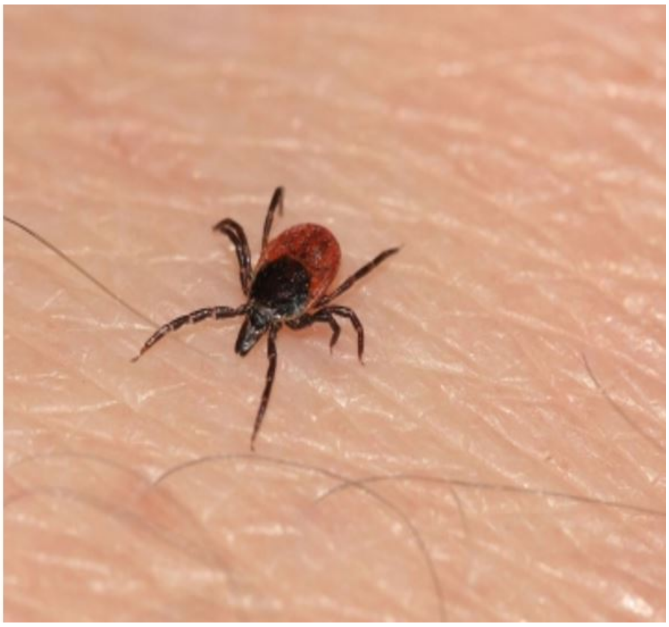

Safety and precautions (3/3)

4-poster devices are registered by the U.S. Environmental Protection Agency (EPA) The EPA requires that pesticide warning signs be placed around each device If a device is placed within 300 feet of a home or place children may be present, a 2 1/2-foot-high fence must surround it Hunter-killed deer that have been treated by a 4-poster device are safe to eat

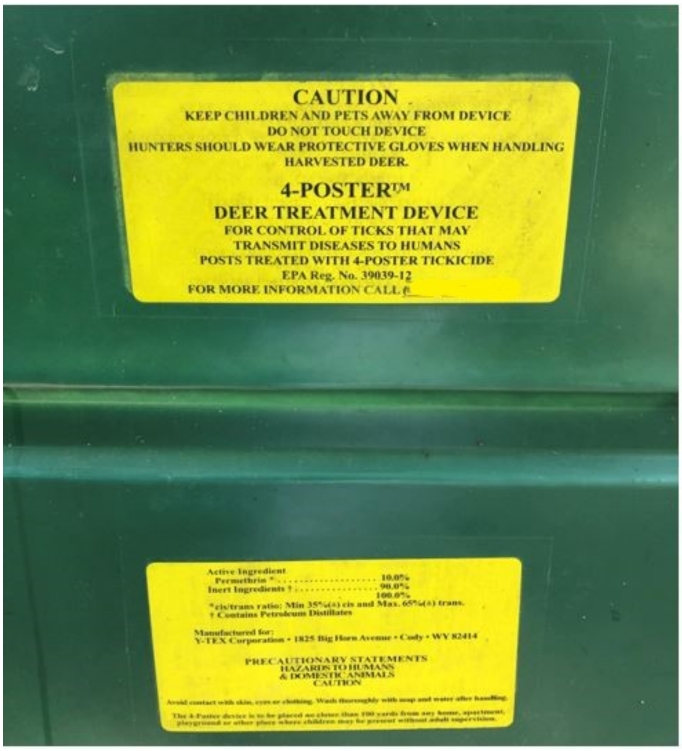

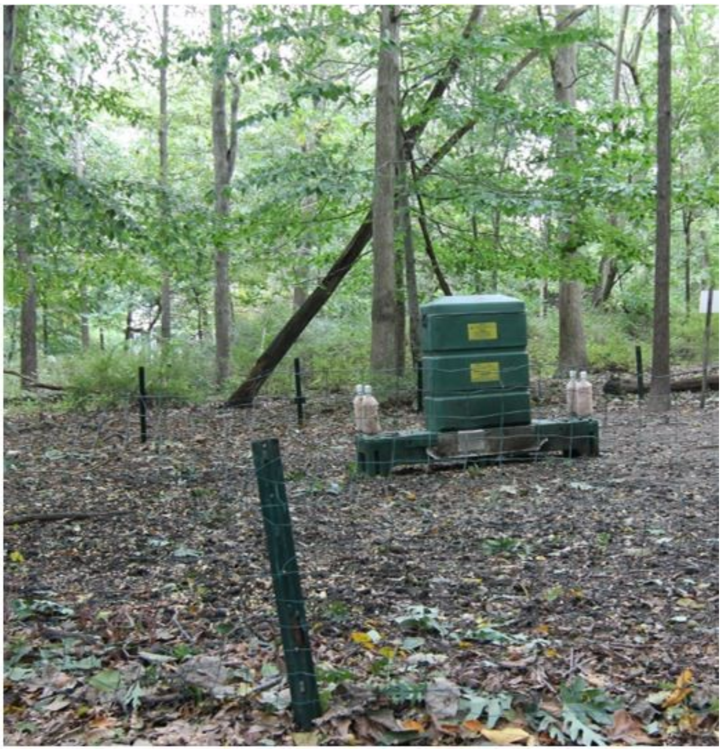

Would you support the placement of a 4-poster device on...

	Yes	No	Unsure	Prefer not to answer
... your own property?	◯	◯	◯	◯
... other private land in your neighborhood or community? (e.g., neighboring homes, commercial properties)	◯	◯	◯	◯
... public land in your neighborhood or community? (e.g., state parks, town parks, other lands owned by municipalities)	◯	◯	◯	◯

Which of the following are reasons that you selected [own_prop] for supporting placement of a 4-poster device ON YOUR PROPERTY?

	Select all that apply
I am not worried about ticks on my property.	☐
I am concerned how the 4-poster device will look.	☐
I am concerned about the safety of 4-poster devices.	☐
I am concerned about the safety of pesticides for myself, family, or pets.	☐
I am concerned about the safety of pesticides for the environment.	☐
I am concerned about the safety of pesticides for deer and other wildlife.	☐
I have safety concerns about attracting wildlife to my property.	☐
I am concerned about property/landscape damage from deer or other wildlife.	☐
I do not want licensed pest management professionals accessing my property weekly.	☐
I use or would rather use other methods of tick control.	☐
I feel my homeowner's association or town would not allow it.	☐
I need more information on how 4-poster devices work.	☐
I do not think this applies to where I live.	☐
Another reason not listed here.	☐
Prefer not to answer	☐

Please list the reason.

____

Which of the following are reasons that you selected [other_priv] for supporting placement of a 4-poster device on OTHER PRIVATE LAND IN YOUR NEIGHBORHOOD OR COMMUNITY?

	Select all that apply
I am not worried about ticks in my neighborhood or community.	☐
I am concerned how the 4-poster device will look.	☐
I am concerned about the safety of 4-poster devices.	☐
I am concerned about the safety of pesticides for myself, family, or pets.	☐
I am concerned about the safety of pesticides for the environment.	☐
I am concerned about the safety of pesticides for deer and other wildlife.	☐
I have safety concerns about attracting wildlife to my neighborhood or community.	☐
I am concerned about property/landscape damage from deer or other wildlife.	☐
I feel my homeowner's association or town would not allow it.	☐
I need more information on how 4-poster devices work.	☐
I do not think this applies to my neighborhood or community.	☐
Another reason not listed here.	☐
Prefer not to answer	☐

Please list the reason.

____

Which of the following are reasons that you selected [public] for supporting placement of a 4-poster device ON PUBLIC LAND?

	Select all that apply
I am not worried about ticks on public lands.	☐
I am concerned how the 4-poster device will look.	☐
I am concerned about the safety of 4-poster devices.	☐
I am concerned about the safety of pesticides for myself, family, or pets.	☐
I am concered about the safety of pesticides for the environment.	☐
I am concerned about the safety of pesticides for deer and other wildlife.	☐
I have safety concerns about attracting wildlife to public lands.	☐
I feel my town would not allow it.	☐
I need more information on how 4-poster devices work.	☐
Another reason not listed here.	☐
Prefer not to answer	☐

Please list the reason.

____

What is your age in years? __________________________________

What is your gender?

- ◯ Female
- ◯ Male
- ◯ Other
- ◯ Prefer not to answer

Do you rent or own your property?

◯ Rent
◯ Own
◯ Prefer not to answer

What size is your property?

- ◯ Less than 0.5 acre
- ◯ 0.5 to 1 acre
- ◯ 1 to 2 acres
- ◯ 2 to 3 acres
- ◯ 3 to 4 acres
- ◯ 4 to 5 acres
- ◯ Greater than 5 acres
- ◯ My property does not have a yard
- ◯ Prefer not to answer

How many people (including children) live in your household? __________________________________

Do children (under the age of 18) live in your household?	◯ Yes<br>◯ No<br>◯ Prefer not to answer

To ensure you receive a gift card for taking this survey, we must confirm your mailing address. Is the address listed on the postcard your mailing address?	◯ Yes<br>◯ No<br>◯ Prefer not to answer<br>(Please select "No" to update your mailing address or select "Prefer not to answer" if you do not want to receive a gift card.)
Please enter the address to which you would like your gift card mailed.	__________________________________

Do you have any additional comments to share with us about community tick control or 4-poster devices?

____

Thank you for your time today! Your responses will help us learn how to better support you in the prevention of tickborne diseases.

Please hit SUBMIT to ensure your responses are recorded and you receive a gift card.

Thank you!
Yale Emerging Infections Program
Phone: 203-764-7949
Email: ticknet@yale.edu
Website: TickNet

You did not meet the eligibility criteria to complete this survey. Thank you for your time.

Please hit SUBMIT to exit the survey.

Thank you!
Yale Emerging Infections Program
Phone: 203-764-7949
Email: ticknet@yale.edu
Website: TickNet
